# Infection control interventions in small rural hospitals with limited resources: results of a cluster-randomized feasibility trial

**DOI:** 10.1186/2047-2994-3-10

**Published:** 2014-03-28

**Authors:** Kurt B Stevenson, Katie Searle, Grace Curry, John M Boyce, Stephan Harbarth, Gregory J Stoddard, Matthew H Samore

**Affiliations:** 1 The Ohio State University Medical Center, N-1122 Doan Hall 410 West 10th Avenue, Columbus, OH 43210, USA; 2 University of Utah School of Medicine, Salt Lake City, UT, USA; 3 Yale-New Haven Hospital, New Haven, CT, USA; 4 Service de Prévention et Contrôle de l'Infection, Hôpitaux Universitaires de Genève, CH-1211 Geneva 14, Switzerland; 5 Informatics, Decision Enhancement, and Analytic Science (IDEAS) Center, VA Salt Lake City Health Care System, Division of Epidemiology, University of Utah School of Medicine, Salt Lake City, UT, USA

**Keywords:** Hand hygiene, Epidemiology, United States, Transmission, Clinical trial, MRSA

## Abstract

**Background:**

There are few reports on the feasibility of conducting successful infection control (IC) interventions in rural community hospitals.

**Methods:**

Ten small rural community hospitals in Idaho and Utah were recruited to participate in a cluster-randomized trial of multidimensional IC interventions to determine their feasibility in the setting of limited resources. Five hospitals were randomized to develop individualized campaigns to promote HH, isolation compliance, and outbreak control. Five hospitals were randomized to continue with current IC practices. Regular blinded observations of hand hygiene (HH) compliance were conducted in all hospitals as the primary outcome measure. Additionally, periodic prevalence studies of patient colonization with resistant pathogens were performed. The 5-months intervention time period was compared to a 4-months baseline period, using a multi-level logistic regression model.

**Results:**

The intervention hospitals implemented a variety of strategies. The estimated average absolute change in “complete HH compliance” in intervention hospitals was 20.1% (range, 7.8% to 35.5%) compared to −3.1% (range −6.3% to 5.9%) in control hospitals (p = 0.001). There was an estimated average absolute change in “any HH compliance” of 28.4% (range 17.8% to 38.2%) in intervention hospitals compared to 0.7% (range −16.7 to 20.7%) in control hospitals (p = 0.010). Active surveillance culturing demonstrated an overall prevalence of MRSA carriage of 9.7%.

**Conclusions:**

A replicable intervention significantly improved hand hygiene as a primary outcome measure despite barriers of geographic distance and lack of experience with study protocols. Active surveillance culturing identified unsuspected reservoirs of MRSA colonization and further promoted IC activity.

## Introduction

Significant investments in biomedical research have been made by the National Institutes of Health (NIH) and other funding agencies. Despite these efforts, much of the research has not “translated” into significant improvements of care at the patient and provider level [1,2]. Two roadblocks to such translational research have been described: the transfer of understanding of disease mechanisms to new methods of diagnosis and therapy (T1 or translation to humans) and the movement of clinical studies into every day practice (T2 or translation to patients) [3-5]. The later phases of translational research (T3 and T4) focus on moving evidence-based practices into health practice and ultimately to population impact [6]. In this study, we examine the feasibility of T3 research in small rural hospitals where limited resources present distinct challenges.

Basic infection control (IC) interventions consist of surveillance, hand antisepsis, and appropriate isolation of patients colonized or infected with multidrug resistant organisms (MDROs) [7-9]. Hand hygiene (HH) is one of the simplest and most effective interventions to prevent the spread of infectious organisms within the healthcare setting [10-12]. Despite its recognized effectiveness, compliance with HH recommendations has been consistently low, typically below 50% [13]. Recently published guidelines have reemphasized the importance of HH and have promoted the use of alcohol-based hand rub for routine hand disinfection [11]. Multidisciplinary approaches to improve HH require resources and commitment on the part of the participating healthcare organization. Thus, most of the HH intervention studies have been conducted in larger, often academic, medical centers [10,14-17]. Rural hospitals have typically been excluded because of small size, low patient census, and remote locations. Furthermore, these smaller facilities often struggle with inadequate financial resources and limited staff making full implementation of HH or infection control interventions appear less feasible [18,19]. There are limited published studies regarding rural IC quality improvement programs or rural healthcare worker (HCW) compliance with IC guidelines [20,21]. Additionally, less is known about rates of transmission of healthcare-associated infections and infection control activities in most rural settings relative to larger facilities [22-24].

Accordingly, this project was undertaken as a feasibility study to test the potential of small rural hospitals with limited resources to evaluate IC interventions according to a standardized research protocol. The research study measured rates of HH compliance as a primary outcome measure of IC compliance in 10 rural community hospitals in Idaho and Utah. We tested, in a prospective, controlled fashion, whether organized, multimodal interventions to improve IC could be successfully implemented. In addition, we screened inpatients routinely with active surveillance cultures to identify reservoirs of resistant pathogens and provided expert IC advice on outbreak control. This provided opportunity to assess the level of colonization within the rural environment and reinforce IC practices.

## Methods

### Setting and study subjects

This study was conducted in a sample of rural hospitals in Idaho and Utah. Hospitals were recruited to participate from a larger pool of rural hospitals in both states already involved in a laboratory surveillance study for resistant pathogens [25]. Inclusion criteria for the current study were: a) hospitals meeting the Office of Management and Budget definition of rural location [26]; b) hospitals indicating the availability of alcohol-based hand gels in their facility; c) hospitals with administrative support for active interventions including inpatient surveillance culturing; and d) hospitals with an infection preventionist (IP) willing to supervise HH compliance monitoring. The selected hospitals were separated by significant geographic distances in both states.

HCWs of all types in each hospital were the object of observation and monitoring of HH compliance. Observers were recruited by the IP from each hospital to conduct blinded observations of HCWs for HH compliance. During the time frames selected to determine the point-prevalence of methicillin-resistant *Staphylococcus aureus* (MRSA) and vancomycin-resistant enterococci (VRE) by active surveillance culturing, those patients selected as candidates for providing surveillance cultures included any inpatient ≥18 years of age capable of giving informed consent. The University of Utah Institutional Review Board (IRB) and Western IRB (Olympia, WA) approved the study; informed consent was required for inpatients providing surveillance cultures.

### Design

The structure of this study was a cluster-randomized trial [27] consisting of a common 4-month baseline measurement period followed by randomization of hospitals into “control” or “intervention” groups and a subsequent intervention time period of 5 months to examine the feasibility of implementing the research protocol. The design of this controlled clinical trial was considered pragmatic in that it addressed effectiveness or the degree of benefit of these infection control interventions in a real practice setting [28]. The study was conducted between March, 2003 and February, 2004.

### HH monitoring

Observers were recruited for each hospital by the IP. IPs from each participating hospital received training from the investigative team in a group session. Each IP was provided with a training manual, a standard data collection form, and uniform definitions for data collection. A training videotape was developed which included 19 scenarios representing different types of HH opportunities. The IPs completed data collection forms while watching the videotape to test inter-rater reliability and resolve areas of disagreement. IPs subsequently trained local observers. Central research staff monitored protocol adherence of local observers during study site visits; however, it was not feasible to measure the inter-rater reliability of local observers across different hospitals.

HH observations were conducted at randomly selected times of the day between 8 am and 5 pm on weekdays in 30-minute increments following established standards for HH observations [10,11]. Variables recorded included type of HCW, nature of patient contact and type of care, contact with the patient care environment, type of hand hygiene (hand washing with soap and water or use of alcohol hand gel), and HH before and/or after patient contact. The number of observed opportunities for hand hygiene was defined as the denominator, and the number of observed opportunities in which hand hygiene was performed served as the numerator. Percent compliance with HH recommendations was calculated. “Complete compliance” was defined as alcohol gel rub or hand washing before *and* after patient/environmental contact; “any compliance” was defined as alcohol gel rub or hand washing before *or* after patient/environmental contact. The duration or efficacy of HH technique was not measured. HH compliance was monitored in all hospitals during the baseline and intervention time periods.

### Intervention components

After the 4-month baseline time period, a hospital-wide campaign to improve compliance with HH and isolation precautions was conducted in each intervention hospital while the control group continued with their usual IC practices. Each hospital staff developed a customized individualized campaign to promote compliance with HH and isolation practices. Elements of this campaign included education sessions, ensuring availability of alcohol-based hand cleaners and personal protective equipment at all patient care areas, written materials, academic detailing, displaying posters that emphasize the importance of hand hygiene and standard precautions, and recognition and rewards programs. The IP of each hospital was instructed to organize the campaign for their hospital.

Results of active surveillance cultures were provided to the intervention hospitals concurrently and to the control hospitals at the conclusion of the study. If the clinical MRSA infection rate exceeded the pre-determined threshold (3 standard deviations above the group mean), the investigative team offered assistance with active infection control interventions to curb further transmission. The following types of additional interventions were considered: education on HH and isolation precautions; campaign to promote isolation of patients; additional surveillance cultures; and decolonization of culture-positive patients [16,29,30].

### Active surveillance cultures

Clinical cultures obtained for clinical indications which yielded MRSA or VRE were continuously reported and analyzed by the hospitals as previously described [25]. Additionally, active surveillance culturing (nasal swabs for MRSA and rectal swabs or stool cultures for VRE or MRSA) on all hospitalized patients ≥ 18 years of age giving informed consent were conducted on four separate days during the study in both groups of hospitals to determine the prevalence of colonization. These were conducted consistently by one member of the investigative team with the assistance of the local IP or trained observer. Swabs were plated for *S. aureus* (blood agar or mannitol salt agar) or enterococcal species (blood agar or bile esculin azide agar). Presence of methicillin or vancomycin resistance was detected by standard methods (conventional MIC testing, oxacillin salt screening agar, or selective media containing vancomycin) [31].

### Statistical methods and data analysis

The primary endpoint was the proportion of health care providers in targeted hospitals compliant with HH protocols (number of compliant observations/total number of observations). This was stratified by “complete compliance” or “any compliance” as outlined above. Secondary endpoints included the rate of clinical cases of MRSA or VRE infection calculated as the number of cases per 1000 patient days and the point prevalence of patients colonized with MRSA and VRE in all participating hospitals as determined by active surveillance culturing (% colonized patients/total patients cultured).

HH compliance data were analyzed using mixed effects logistic regression to model the probability of compliance. These models accounted for the lack of independence introduced by the individual hand hygiene opportunities being nested within hospital. The study group indicator variable, intervention relative to non-intervention, and intervention period indicator variable, post-intervention relative to pre-intervention, were included as main effect terms. The interaction term, study group × intervention period, was used to statistically test the null hypothesis that the change in probability of compliance was equal in the two study groups. To provide a clear presentation of the results, both group and hospital-specific predicted probability of compliance was estimated from the models for both the pre- and post-intervention periods, and then subtracted to measure the absolute change in compliance. This allowed communication of the results in the more readily understood terms of absolute percent change rather than odds ratios.

## Results

### Study hospitals

Ten rural hospitals in diverse geographic locations in Idaho and Utah were recruited to participate in this study performed in 2003. Five hospitals were each randomized to the intervention or control arms of the study. One control hospital elected to withdraw from the study shortly after the intervention time period had begun and was not included in the final analysis. The mean bed size of the intervention hospitals (62 licensed beds) was not significantly different from the control group (44 licensed beds, p = 0.62). Four of the five intervention hospitals reported that they had little awareness of the magnitude of MRSA cases or the prevalence of colonized patients in their facilities prior to initiation of this study.

### Hand hygiene component

Each intervention hospital was provided guidance and a budget to develop a customized campaign to improve infection control practices in their facility. All campaigns focused on hand hygiene and were instituted by each intervention hospital during the 5-month intervention period. As shown in Table 1, most hospitals provided group education with a number of approaches (lectures, videos, demonstrations, displays, games, tests).

**Table 1:** Summary of Interventions Employed in the Intervention Hospitals During the Study

**Hospital**	**Bed size**	**Group education**	**Education type**	**Posters**	Poster audit*	**Poster rotations**	**Rewards**	**Buttons**	**Ink pens**	**Back scratchers**	**Candy bars**
1	38	Yes, 12 sessions	games, demonstration, feedback, videos, displays, testing, handouts	Yes	22	unknown	buttons, ink pens, backscratchers, candy, food, other prizes	140	251**	80	51*
2	21	Yes, 4 sessions	lectures, feedback of compliance data	Yes	20	rotated once	buttons, ink pens, backscratchers, candy, food	105	154**	55**	44
3	20	No, one-on-one only	acadmic detailing of individual staff	Yes	20	unknown		36	0	36	0
4	183	Yes, 6 sessions	games, videos, feedback, poster contest	Yes	32	unknown	ink pens, backscratchers, candy, food	300***	950	450**	290**
5	50	Yes, 4 sessions	feedback of compliance data	Yes	18	every 1–2 wks	buttons, ink pens, backscratchers, candy, food/luncheon, GlowGerm	287	280	135	96**

*Investigative team audit of the number of posters hanging in the hospital during visit.

**Most popular items at individual hospital.

***These buttons were recycled to other hospitals.

**Figure 1:**
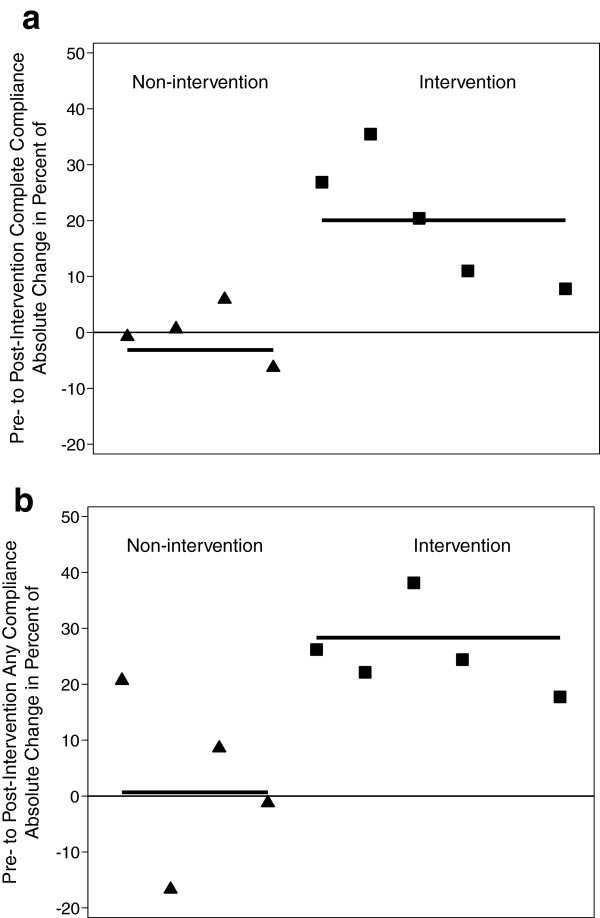
**Absolute changes in probability of (a) “complete compliance” and (b) “any compliance” of hand hygiene during the pre-intervention compared to post-intervention period. a** Absolute changes in probability of “complete compliance” of hand hygiene during the pre-intervention compared to post-intervention period. **b** Absolute changes in probability of “any compliance” of hand hygiene during the pre-intervention compared to post-intervention period.

A total of 2,654 hand hygiene opportunities were observed in the five intervention hospitals and 1,873 in the four control hospitals. The two study arms were well-balanced with respect to the percent of opportunities observed across HCW occupations (Table 2). The estimated absolute change in “complete” HH adherence ranged from 7.8% to 35.5% in intervention hospitals and −6.3% to 5.9% in control hospitals. The average absolute change in intervention hospitals was 20.1% in intervention hospitals compared to −3.1% in control hospitals (p = 0.001) (Figure 1a). The estimated absolute change in “any compliance” ranged from 17.8% to 38.2% in intervention hospitals and −16.7 to 20.7% in control hospitals, with an average absolute change of 28.4% in intervention hospitals compared to 0.7% in control hospitals (p = 0.01) (Figure 1b).

**Table 2:** Number of hand hygiene opportunities observed by healthcare worker occupation

**Occupation**	**Nonintervention**	**Intervention**
	**[N = 1,873]**	**[N = 2,654]**
Doctor (MD), n (%)	187 (10)	232 (9)
Nurse (RN)	934 (50)	1,328 (50)
Nurse assistant (CNA)	219 (12)	299 (11)
Respiratory therapist	50 (3)	72 (3)
Physical therapist	49 (3)	65 (2)
Radiation technician	87 (5)	76 (3)
Laboratory technician	82 (4)	137 (5)
Environmental services	72 (4)	93 (4)
Other	193 (10)	352 (13)

### Surveillance cultures

Among the 9 participating hospitals, 290 unique surveillance cultures were performed during the total 9 month study time period. Among these were 171 nasal swabs alone (rectal swabs being refused by these patients) and 119 instances where both a nasal and rectal swabs were obtained from the same patient. The prevalence of MRSA carriage was 28 of 290 (9.7%) and of VRE carriage was 1 of 119 (0.84%). Rectal swab cultures detected three MRSA carriers not detected by nasal swab. The median number of unique positive cultures of MRSA per hospital was two (range: 0 to 11). As results of active surveillance cultures were communicated, two of the smaller hospitals initiated new programs to identify previously infected or colonized MRSA patients upon readmission. One hospital identified a local long-term care facility (LTCF) as a source of MRSA cases in their hospital and provided education resources to the LTCF.

### Rural facility MRSA cluster

**Figure 2:**
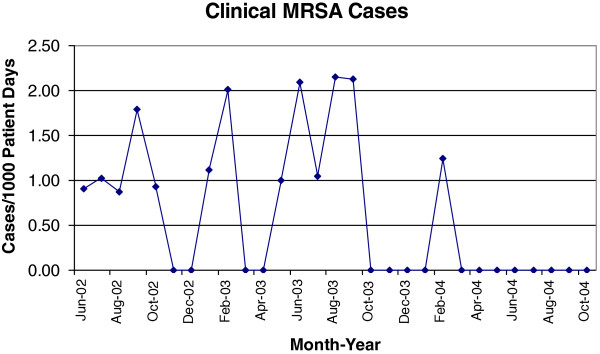
**Rates of methicillin-resistant *Staphylococcus aureus *** **infection clinical cases in one selected intervention hospital.**

The results of one specific intervention are highlighted to illustrate the impact of investigator interaction with the participating facilities on IC practices. One intervention hospital with an attached long-term care unit (LTCU) had a noticeably high number of positive MRSA surveillance cultures and was the focus of targeted infection control interventions. This hospital was very small with an average daily census of 5 patients. There was a cluster of positive surveillance cultures initially noted in June 2003 (n = 5) with 4 cultures from residents of the LTCU. This number of positive cultures exceeded the predetermined threshold of 3 standard deviations above the mean for all participating hospitals. Surveillance cultures were repeated in the acute care and long-term care unit in July 2003 with 3 positive cultures noted in new patients and 1 positive culture from a patient that had been previously negative in June 2003. Interventions began in August 2003 with increased education, repeating of active surveillance cultures, selective de-colonization of all patients with new and current MRSA colonization, enhanced HH, and more aggressive application of contact precautions. Decolonization of all residents with positive surveillance cultures was performed by implementing bathing with chlorhexidine soap, application of mupirocin ointment to anterior nares, and oral antimicrobial therapy (trimethoprim/sulfamethoxazole and rifampin) according to published protocols [30]. These decolonization interventions were applied for 7 days with follow-up surveillance cultures in 4–6 weeks. Repeat surveillance cultures in November 2003 revealed no further colonized patients. There was only one patient with MRSA infection identified in the next 12 months and this was identified upon admission and not associated with healthcare transmission (Figure 2).

## Discussion

We performed a rigorously designed cluster-randomized trial to implement IC interventions in small rural hospitals in the western United States. IC interventions designed to prevent transmission of resistant pathogens, as demonstrated here, are feasible in small rural hospitals. Utilizing existing hospital staff, all hospitals were able to follow a research protocol to measure HH compliance as a primary outcome measure. Additionally, intervention hospitals were able to design and implement effective campaigns to enhance compliance with IC practices. This study outlines a replicable intervention which significantly improved HH and IC practices in rural hospitals despite geographic distance and lack of experience with study protocols. This study differs from many other HH studies in that it was designed as a cluster-randomized trial rather than a before and after measurement. It also illustrates that patients in these settings are reasonably receptive to performing active surveillance cultures when informed that they are part of a hospital quality improvement and infection control program. The impact of sustainable hand hygiene and infection control interventions on the transmission of healthcare-associated infections in the rural setting is a subject for future intervention studies.

As reported by intervention hospitals, active surveillance culturing for MRSA and monitoring of clinical MRSA cases promoted the implementation of HH and isolation guidelines. The primary endpoint of this study was measuring compliance with HH, not isolation precautions, or monitoring actual transmission. These other endpoints could be targeted in future studies. As illustrated in the example from one rural hospital, intensive IC efforts consisting of active surveillance culturing, implementing contact isolation precautions, and decolonization of colonized patients were feasible and may be effective in reducing MRSA cases and healthcare transmission in the rural setting. Further studies are needed to substantiate these preliminary observations. The success of such interventions has been demonstrated in larger hospitals [16,29,32-34] but there remains little reported experience in rural hospitals [20,21]. Linking small hospitals with larger facilities capable of providing infection control expertise and resources, as demonstrated in this study, may be also be a feasible model for future evaluation. The unique characteristics of rural hospitals warrant the evaluation of such new approaches to infection control and antimicrobial resistance management [22].

Studies from our investigative group have demonstrated that, when present in a rural community, the incidence of MRSA clinical infections in rural hospitals is similar to that seen in larger urban centers [25]. Although the presence of MRSA was variable and not detectable in all rural communities evaluated, the present study confirms that MRSA carriage is present in the majority of rural community hospitals and may even be higher now given the increase in community-onset MRSA [35,36]. VRE, however, was rarely found on surveillance cultures substantiating our recent observation that VRE was uncommon as a clinical isolate in a large cohort of rural hospitals [25]. The prevalence of MRSA, however, appears significant enough to consider consistently implementing routine HH and the appropriate isolation precautions to prevent MRSA transmission in these small hospitals.

This study has several potential limitations. Although cluster-randomized, this study involved a small number of hospitals in a select region of the Western US. It is uncertain if these results can be generalized to all US rural hospitals. One control hospital withdrew from the study early which may have influenced the overall outcomes in the control group. The intervention time period was only five months and was designed to assess feasibility of such interventions. This study does not address, therefore, the sustainability of the IC and HH interventions over time or its long-term impact on reducing healthcare transmission of MRSA or other resistant pathogens. These outcomes are the subject for future intervention studies. Although HH behavior was directly observed, no quantitative assessment of the level of HH (soap or alcohol-based hand rub consumption) was performed.

In summary, a replicable intervention which significantly improved HH in rural hospitals is demonstrated along with an illustration of the feasibility of implementing aggressive infection control interventions to reduce healthcare-associated transmission of significant pathogens in these small hospitals. Future studies to evaluate the sustainability of HH and infection control measures and their long-term impact on transmission rates in this group of small hospitals with limited resources are needed.

## Competing interests

None of the authors discloses any financial or other conflicts associated with this study.

## Authors’ contributions

KBS: conception of study and study design; on site implementation of study; data acquisition; data analysis; drafting of the manuscript. KS: on site implementation of study; data acquisition; critical review of the manuscript. GC: on site implementation of study; data acquisition; critical review of the manuscript. JB: conception of study and study design; consultative support; data analysis; critical review of the manuscript. SH: conception of study and study design; a consultative support; data analysis; critical review of the manuscript. GS: conception of study and study design; data analysis; statistical analysis; critical review of the manuscript. MS: conception of study and study design; supervision and on site implementation of study; data acquisition; data analysis; statistical analysis; obtained funding; critical review of the manuscript. The manuscript was reviewed and approved by the authors.
